# Endoplasmic reticulum stress-mediated membrane expression of CRT/ERp57 induces immunogenic apoptosis in drug-resistant endometrial cancer cells

**DOI:** 10.18632/oncotarget.17678

**Published:** 2017-05-08

**Authors:** Qin Xu, Chuanben Chen, An Lin, Yunqing Xie

**Affiliations:** ^1^ Department of Gynecological Oncology, Fujian Cancer Hospital, Teaching Hospital of Fujian Medical University, Fuzhou, China; ^2^ Department of Oncology, Fujian Cancer Hospital, Teaching Hospital of Fujian Medical University, Fuzhou, China; ^3^ Department of Research Center, Fujian Cancer Hospital, Teaching Hospital of Fujian Medical University, Fuzhou, China

**Keywords:** doxorubicin, endometrial carcinoma, calreticulin, endoplasmic reticulum stress response, drug tolerance

## Abstract

**Objective:**

To investigate the role of endoplasmic reticulum (ER) stress-mediated CRT/ERp57 complex expression underlying the mechanism of resistance to doxorubicin (DOX) in endometrial carcinoma (EC) *in vivo* and *in vitro*.

**Methods:**

The expression of CRT, ERp57, p-PERK, eIF2α, p-eIF2α in EC patients and EC cells was detected by Western blots and by immunofluorescence assay. MTT assay was used to determine the LC50 of EC cells to DOX and cell viability. Apoptosis was assayed using flow cytometer. The protein expression of PERK, cleaved-caspase-8, p-eIF2α and CHOP were detected by Western blot, and the expression of VAMP-1, SNAP23 and PERK was knockdown by siRNA and/or shRNA. The expression of CRT/ERp57 complex was detected by flow cytometry. In addition, the expression of eIF2α and p-eIF2α was detected by Western blot analysis after drug-resistant EC cells were transfected with lentivirus overexpressing CRT, treated with GADD34 inhibitor and ES stress inducer. MTT assay was used to detect the phagocytic activity of T cells induced by maturation of dendritic cells in drug-resistant EC cells.

**Results:**

The expression of CRT, ERp57, p-PERK and p-eIF2α was significantly decreased in the drug-resistant patients in EC patients. The IC_50_ of the drug-resistant EC cells was 10 times higher than that of the wild type cells. In the drug-resistant EC cells the expression of CRT, ERp57, p-PERK, p-eIF2α, caspase-8 and CHOP was significantly lower than in the wild type cells. After treatment with DOX, CRT and ER stress-related proteins p-PERK, p-eIF2α, caspase-8 and apoptosis were significantly increased in wild-type EC cells, but not in drug-resistant EC cells. The increased expressions led to inhibition of cell growth and apoptosis. The knockdown of PERK gene and addition of DOX resulted in significant decrease of cleaved-caspase 8 and p-eIF2α in sensitive EC cells. The expression of CRT/ERp57 in sensitive EC cells was further significantly decreased by blocking VAMP and SNAP23. In addition, transfection with CRT overexpressing lentivirus and addition of GADD34 inhibitor and ER stress inducer in drug-resistant EC cells revealed a significant increase in the expression of CRT/ERp57 complex and p-eIF2α when DOX was added simultaneously, which promoted the maturation and chemotaxis of T lymphocytes to phagocytose drug-resistant EC cells.

**Conclusion:**

DOX can induce the death of tumor cells by ER stress-mediated CRT/ERp57 expression in EC cells. Induction of ER stress in drug-resistant EC cells up-regulates the membrane expression of CRT/ERp57, enhances phagocytosis, induces immunogenic apoptosisand sensitizes the cells to DOX.

## INTRODUCTION

Endometrial carcinoma (EC) is a common malignant cancer in the female reproductive systems that occurs in the endometrial epithelium of genital tract. It accounts for 20 to 30% of the malignant tumors in female genital tract [1]. In recent 5 years, the incidence of this disease has increased in the world, while the 5-year survival rate has decreased. Although a number of treatments are available for the cancer, such as surgery, chemotherapy, radiotherapy and endocrine therapy, chemotherapy is becoming increasingly important for clinical treatment of EC, especially for advanced and drug-resistant EC.

Studies have shown that due to unknown reasons or diseases that cause calcium imbalance or disorder in processing and transport of proteins, endoplasmic reticulum (ER) becomes dysfunctions and could result in ER stress and apoptosis, apoptosis resistance is an important characteristic of drug resistance [2–4]. Among the proteins, calraticulin (CRT) is an ER binding protein that regulates the calcium homeostasis [5–8]. Study found that the expression of CRT is regulated by ERP57 (a member of disulfide isomerase family) [9]. Calreticulin and ERP57 is translocated in the same molecular complex. Knockout of ERp57 is shown to inhibit the expression of CRT and maturation of dendritic cells (DCs) and induced phagocytosis, resulting in loss of immunogenicity *in vivo*. Through gene knockout and other methods, CRT expression could be deprived absent to reduce ERp57expression of [10]. ERp57 knockdown tumor cells may be induced to generate immune response by chemotherapeutic drugs after the cells are added with recombinant CRT. CHOP is also called growth arrest and DNA damage-inducible protein 153 (GADD153). It is an ER-specific stress transconductor and belongs to the CCAAT/enhancer-binding protein (C/EBP) transcription factor family [11]. Recent studies show that CHOP is normally located in the cytoplasm at very low level. However, during stress, its expression is up-regulated via the IRE1-XBP1 and ATF6 pathways. In addition, the PERK-eIF2α pathway selectively upregulates ATF4, which activate CHOP and other genes in amino acid metabolism, redox reaction and transport, leading to significantly increase in the expression of CHOP [12].

Doxorubicin (DOX) is a member of anthracycline drugs that are shown to induce ER-mediated transmembrane translocation of CRT and release of ATP through autophagy, resulting in immunogenic apoptosis [13]. However, some patients are insensitive to chemotherapy, or may develop resistance to chemotherapy after treatment. Previous study shows that in DOX-resistant colon cancer cell lines, the membrane expression of CRT was decreased significantly, indicating that the expression is related to DOX resistance. Approaches that increase the expression may overcome the tolerance [14]. Currently, combination of DOX and carboplatin is still the first-line chemotherapy regimen for EC. However, the model of action of DOX in EC remains largely unknown. Therefore, this study was aimed to investigate the molecular mechanism of DOX in EC *in vivo* and *in vitro* and impact of relevant pathways on DOX tolerance. The findings would provide new insights for overcoming DOX tolerance for better EC treatment.

## RESULTS

### Patient information

In our cohort, we included 25 endometrial cancer samples. The age of patients ranged 34–69 years, with an average age of 55 years. The stage were: 32% stage II, 48% stage III and 20% stage IV. The history of tumor were: 80% endometrioid and 20% non-endometrioid. Tumor sizes were: less than 4 cm in 12% of the samples, and larger than 4 cm in 88%. Metastatic lymph nodes were found in 60% of patients. The menopausal state were: 24% pre-menopausal and 76% post-menopausal. The percentages of tumor grading were: 36% grade III and 64% grade I–II. The sensitive to the NAC were: 40% sensitive and 60% resistant (Table 1).

**Table 1:** Patient information

Clinical features		No. patients
Age	>45y	18
	≤ 45y	7
Stage	II	8
	III-IV	17
History	Endometrioid	20
	Non-endometrioid	5
Tumor size	>4cm	22
	≤ 4cm	3
Lymphnode metastasis	Positive	15
	Negative	10
Differentiation	G1-G2	16
	G3	9
NAC	Sensitive	10
	Resistant	15

NAC: neoadjuvant.

### Expression of the CRT pathway in EC patients

**Figure 1:**
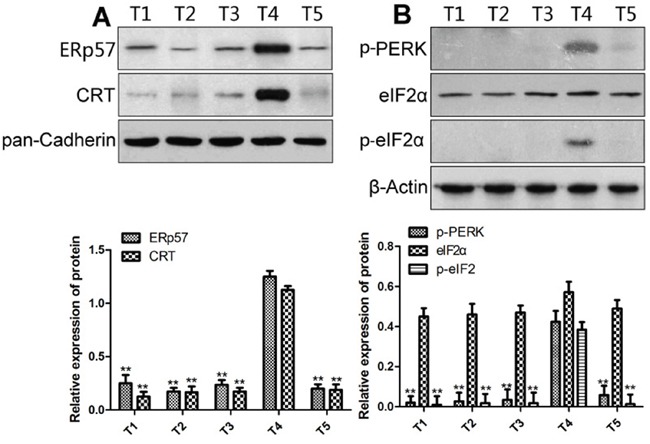
The membrane expression of CRT **(A)** and ER stress-related proteins **(B)** in EC cells from drug-sensitive and resistant patients. Upper panels, representative Western blots; lower panels, relative protein levels. * and ** denote significantly difference vs T4 patient at *P* < 0.05 and 0.01 levels, respectively.

Retrospective analysis of the patients admitted to Fujian cancer hospital in 2013 showed that the patients had different levels of DOX tolerance. In comparison with sensitive patients (T4), CRT and ERp57 expression in resistant patients (T1, 2, 3 and 5) were significantly reduced in the EC cells (Figure 1A, *P* <0.05). In addition, the expression of p-PERK and p-eIF2α was also significantly decreased (Figure 1B, *P* <0.05). These data suggested that the membrane expression of CRT was significantly reduced and the PERK/eIF2α was inactivated in DOX-resistant EC patients as compared with the sensitive EC patients.

### DOX resistance of EC cell lines

**Figure 2:**
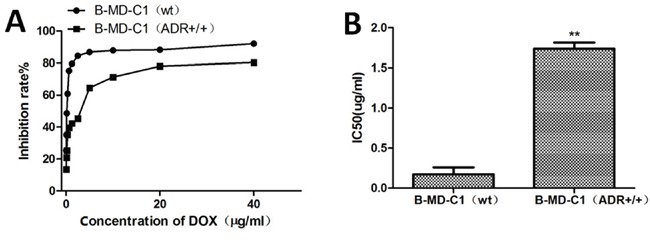
Growth **(A)** and LCs_50_ **(B)** of drug-sensitive and resistant EC cells in DOX-supplemented medium.

The LCs50 of wild-type and DOX-resistant EC cell line B-MD-C1 (ADR+/+) were 0.17 and 1.737 μg/ml DOX, respectively (Figure 2A, 2B), the latter was significantly greater than the former (*p* < 0.01).

### Effect of DOX on ER stress and the expression of CRT/ERp57 in EC cells

**Figure 3:**
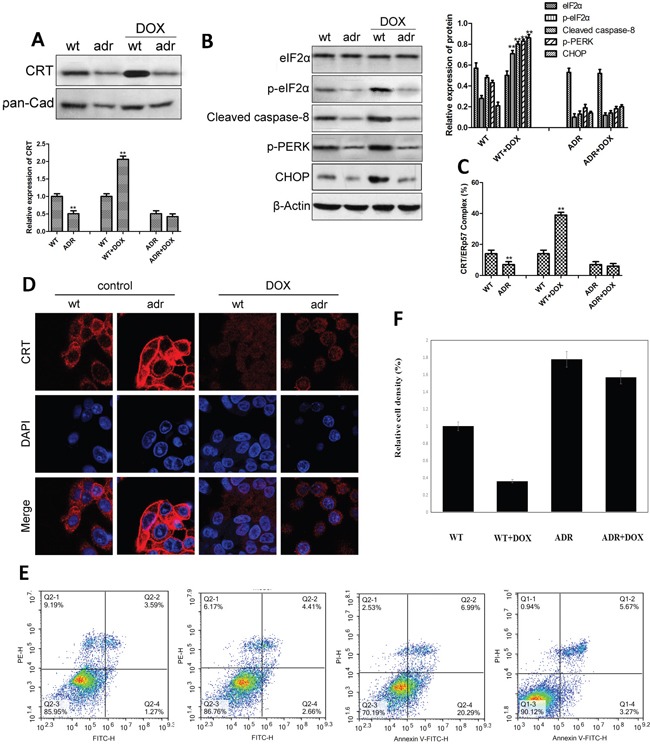
The expression of CRT and ER stress-related proteins and apoptosis in the wild-type and drug-resistant EC cells following DOX treatment (**A** and **B**) Upper panes: representative Western blots, low panes: relative protein levels in absence and presence of DOX. **(C)** Expression of CRT/ERp57 assayed using flow cytometry. **(D)** Expression of CRT assayed using immunofluorescence. **(E)** Cytometry assay of apoptosis. * and ** denote significantly difference vs control/wt at *P* < 0.05 and 0.01 levels, respectively.

We then investigated the effect of DOX on ER stress and the expression of CRT/ERp57 in EC cells. The results showed that the expression of CRT was significantly lower in drug-resistant EC cells than in drug-sensitive wt cells (Figure 3A). After DOX treatment, however, CRT was significantly up-regulated in wt cells but not in drug-resistant cells. Compared with wt cells, drug-resistant cells had significantly reduced levels of p-eIF2α, p-PERK, cleaved-caspase-8 and CHOP. Following DOX treatment, again, the expression levels of the three genes were up-regulated only in wt cells but not in drug-resistant cells (Figure 3B). Flow cytometry studies also showed that after DOX treatment, the expression of CRT/ERp57 complex was significantly up-regulated in wt cells (Figure 3C). In comparison with wt cells, the level of CRT/ERp57 was lower in drug-resistant cells, which did not change much after DOX treatment. Similarly, immunofluorescence assays showed that the expression of CRT was significantly up-regulated in wt EC cells but not in drug-resistant cells (Figure 3D). Cytometry assays showed that apoptosis was significantly increased in the wt cells but not in the resistant cells after DOX treatment (Figure 3E). MTT assays indicated that DOX inhibited growth of wt cells but not drug-resistant cells. These data indicated that in EC cells, DOX could activate ER stress to up-regulate the expression of CRT/ERp57 in B-MD-C1 (wt), resulting in apoptosis, while in DOX-resistant cells, the expression of CRT/ERp57 in response to ER stress was reduced, resulting in inhibition of apoptosis.

### Effect of DOX on wt EC cells after inactivation of the ER stress pathway

**Figure 4:**
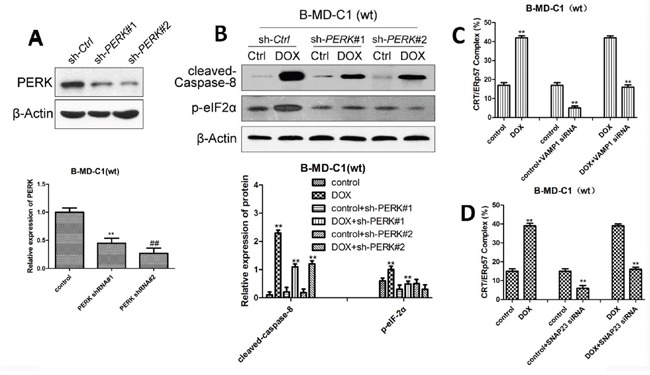
The expression of ER stress-related proteins in wt EC cells following DOX treatment and PERK pathway knockdown alone or in combination (**A** and **B**) Upper panes: representative Western blots, low panels: relative ER stress-related protein levels following knockdown of PERK by shRNA. **(C)** Expression of CRT/ERp57 following knockdown of VAMP1 by siRNA using flow cytometry. **(D)** Expression of CRT following knockdown of SNAP23 by siRNA using immunofluorescence. * and ** denote significantly difference vs control at *P* < 0.05 and 0.01 levels, respectively.

In the above mentioned studies, we found that DOX impact the ER stress pathway in wt EC cells. To further investigate the effect of DOX on drug-sensitive EC cells, we examined if knockdown of the ER stress pathway would impact DOX on CRT/ERp57. Our results showed when PERK was blocked by shRNA, the expression of PERK in EC cells was significantly down-regulated, particularly by PERK#2 sh-RNA (Figure 4A); knockdown of PERK also resulted in decreased level of cleaved-caspase8 and p-eIF2α in wt EC cells (Figure 4B). Compared with DOX treatment alone, PERK knockdown and DOX treatment further reduced the expression of cleaved-caspase8 and p-eIF2α (Figure 4B). When VAMP1 or SNAP23 was knockdown by siRNA, the expression of CRT/ERp57 was down-regulated if DOX was added (Figure 4C, 4D). These results indicated that the inactivation of the ER stress signal pathway in drug-sensitive EC cells would inhibit DOX-induced CRT/ERp57 expression and reduce apoptosis.

### Effect of CRT/ERp57 on DOX activity in drug-resistant EC cells

**Figure 5:**
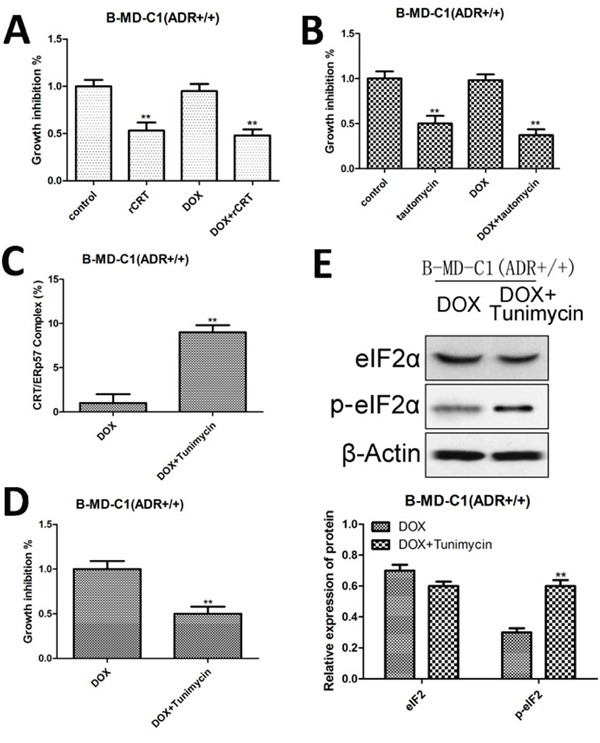
Growth inhibition, CRT/ERp57 and p-elF2α expression in drug-resistant EC cells following overexpressing CRT and treated with tautomycin and tunicamycin (**A** and **B**) Growth inhibition following overexpressing CRT and addition of GADD34 inhibitor (tautomycin). **(C)** Expression of CRT/ERp57 by flow cytometry. **(D)** Growth inhibition following addition of DOX and tunicamycin. **(E)** Upper panel, representative Western blots, low panel: relative protein levels of elF2α and p-elF2α. * and ** denote significantly difference vs control at *P* < 0.05 and 0.01 levels, respectively.

GADD34 protein phosphatase (GADD34) inhibits the phosphorylation of eukaryotic initiation factor elF2a, while GADD34 inhibitor can induce CRT to express rapidly. When CRT was overexpressed or GADD34 inhibitor (tautomycin) was added in B-MD-C1 (ADR+/+), and co-cultured with DCs, the viability of the cells was reduced significantly (Figure 5A, 5B), suggesting that these treatments promote the maturation of DCs for better chemotaxis that induces the phagocytosis of drug-resistant EC cells by T cells. When ER stress inducer tunicamycin was added to drug-resistant EC cells, the surface expression of CRT/ERp57 was up-regulated (Figure 5C); after co-culture with DCs, the cell viability was reduced (Figure 5D), indicating that tunicamycin promotes the maturation of DCs to induce the phagocytosis of drug-resistant EC cells by T cells. In addition, tunicamycin also up-regulated the membrane expression of p-eIF2α in drug-resistant EC cell B-MD-C1 (ADR+/+) (Figure 5E), suggesting that the activation of ER stress signaling pathways may sensitize drug-resistant EC cells to DOX, up-regulate the expression of CRT/ERp57, resulting in immunogenic apoptosis.

### Mechanisms underlying the immunogenic apoptosis induced by ER stress-mediated membrane expression of CRT/ERp57 in drug-resistant EC cells

**Figure 6:**
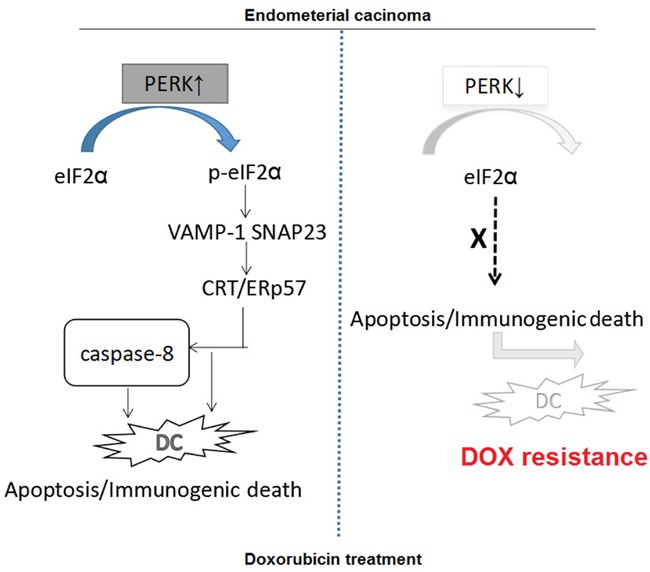
Proposed molecular mechanisms underlying the immunogenic apoptosis induced by ER stress-mediated membrane expression of CRT/ERp57

As shown above that the expression of CRT/ERp57 and ER stress-related proteins was different between drug-sensitive and resistant EC cells. In the drug-sensitive EC cells, DOX could activate the PERK/eIF2a pathway to induce ER stress response, resulting in presentation of CRT/ERp57 from ER to the Golgi apparatus via mediation of VAMP1 and SNAP23 to induce immunogenic apoptosis. On contrast, in the drug-resistant cells, DOX did not down-regulate the expression of PERK in the drug-resistant EC cells nor activate the PERK/eIF2a pathway to induce the expression of CRT/ERp57, nor to induce the expression of downstream caspase-8 to induce immunogenic apoptosis. We found that DOX can effectively induce ER stress in wt EC cells to assist the presentation of CRT/ERp57 complex on cell surface to generate immunogenic apoptosis of the target cells. On the other side, in the drug-resistant cells, the induction of CRT/ERp57 expression in response to ER stress was markedly reduced, resulting in the suppression of immunogenic apoptosis pathway. This could be a new therapeutic target for drug-resistant EC (Figure 6).

## DISCUSSION

The first pathway activated by ER stress response is the most important PERK/eIF2α pathway which activates the expression of CHOP [15]. Over-expression and targeted mutagenesis of CHOP show that CHOP may promote apoptosis during ER stress [16]. As an ER stress-specific apoptogenic factor, CHOP widely exists in many types of cells at low level in normal conditions, and is significantly up-regulated when the cells are stressed [16]. Studies have shown that when ER stress occurs, phosphorylated eIF2α is increased, resulting in transient reduction of globulin synthesis and translational up-regulation of ER stress-related mRNA. If not relieved or the stimulation is persistent, unfolded proteins would initiate the process of apoptosis [17]. Our results showed that in drug-sensitive EC cells, DOX can activate EC stress response, resulting in increased expression of p-PERK, p-eIF2α and CHOP, inhibition of cell growth and apoptosis of the cells.

Loss of expression of tumor antigens and immune tolerance to tumor are the leading causes making tumor not recognized and attached by the host. For chemotherapy, the drugs may induce immunogenic death in three ways: the translocation of CRT to the surface of cell membrane to activate the “eat me” pathway in DCs, the secretion of ATP from dead tumor cells to stimulate phagocytosis and induction of innate immune by HMGB1 from the dead tumor cells [13]. Studies show that disorders in calcium homeostasis or protein processing and transport may result in ER dysfunction and subsequently ER stress [18]. CRT is an ER binding protein, which participates in the regulation of calcium homeostasis [19]. CRT is also involved in “quality control” in ER to prevent protein mis-folding and the aggregation and secretion of incompletely translated human leukocyte antigen (HLA-I) [20, 21]. In addition, when apoptosis is induced in tumor cells, CRT is quickly translocated from ER to the surface of cell membrane, leading to immunogenic death of tumor cell. However, in DOX-resistant cells, the membrane expression of CRT is significantly decreased [20]. The expression of CRT in tumor tissues such as liver, stomach and colon cancers is higher than in normal tissues [22] as well as in urinary system tumors. CRT has been proposed as a potential biomarker for bladder cancer [14, 23, 24]. In our previous study, CRT level was found higher in patients with drug-sensitive EC and lower in patients with drug-resistant EC. Therefore, the expression level of CRT be used as biomarker for the survival and prognosis of EC patients. The findings from the present study confirm that the expression of CRT is significantly lower in drug-resistant than in drug-sensitive EC cells, suggesting that this may be a new therapeutic target for EC.

DCs are important antigen-presenting cells. They initiate specific immune response. In colon cancer cells, anthracyclines can induce the rapid translocation of CRT to the cell surface to cause immunogenic death of the tumor cells [25, 26]. Recently, Panaretakis found that anthracycline drugs mitoxantrone and oxaliplatin can stimulate ER stress in colon cancer cell line CT26, activate the phosphorylation kinase of ER elF2α, resulting in the phosphorylation of elF2α and structural changes of caspase-8, apoptosis regulators BAX and Bak. As a result, CRT already translocated into the Golgi was excluded from the cells via SNARE-dependent exocytosis in form of CRT/ERp57 to exert translocation effect [27]. Recent study shows that DOX may be presented through CRT, HSP70 and HSP90 to cell membrane and the secretion of ATP can induce immunogenic tumor cell death [28]. In addition, in colon cancer cells DOX can induce immunogenic apoptosis by generating nitric oxide to promote the membrane expression of CRT [29]. When eIF2α is mutated (unable to phosphorylate), CRT is unable to translocate successfully to the cell membrane to induce apoptosis [30], indicating that ER stress can mediate CRT translocation. Our study showed that DOX induces immunogenic apoptosis in EC cells via ER stress-mediated translocation of CRT. However, in drug-resistant EC cells, the expression of ER stress-related protein was not activated. Further studies are needed to elucidate the mechanism underlying the difference. In addition, CRT, GADD34 inhibitor and ERS inhibitor (tunicamycin) were found to be able to activate ER stress pathway and induce immunogenic apoptosis in EC cells.

Tumor cell recognition by DCs and presentation of tumor antigen are important to trigger T cell-dependent antitumor immune [31]. Recent studies have found that some pro apoptotic drugs can not only directly damage tumor cells, but also induce tumor cell to display immunogenic molecules such as CRT, promote the maturation of DC to activate tumor antigen-specific T cells and reconstitute specific anti-tumor immune in tumor microenvironment. In recent years, cytotoxic drugs have been found to be able to induce apoptosis in cancer cells and promote the expression of molecules recognizable by DCs such as CRT and HSP70. Through interaction with the DC ligands on cell surface, phagocytosis and tumor antigen presentation of DC are enhanced. This results in the formation of specifically sensitized lymphocytes with reconstructed immune pattern within tumor [32]. When CRT was knockout, immune responses such as the activation of DC by apoptotic cells also disappeared [33]. Therefore, the membrane translocation of CRT can enhance the immunogenicity of dead cancer cells. We showed that DOX could not only induce apoptosis of EC cells but also up-regulate the expression of CRT on the membrane surface to promote the phagocytosis by DC, leading to immunogenic tumor death.

In summary, we demonstrate that membrane expression of CRT in EC cells is closely related to PERK pathway-associated ER stress. The molecular mode of action of DOX is likely to activate ER stress-mediated CRT expression pathway to induce immunogenic apoptosis in EC cells. The findings of this study can be used for the clinical treatment: measurement of CRT levels and determination of the sensitivity to DOX before chemotherapy would help better planning of therapeutic scheme for effective treatment of EC.

## MATERIALS AND METHODS

### Reagents

B-MD-C1 (wt) was purchased from ATCC, DOX (Adriamycin, ADR) was purchased from Hisun pharmaceuticals, Zhejiang, China and dissolved in sterile saline solution (2 mg/ml) and stored at -20 °C before use. Fetal bovine serum (FBS) and RPMI 1640 medium were purchased from Gibco, USA; Lipofectamine™2000 was purchased from Life Technologies, USA; antibodies against CRT, ERp57, caspase-8 and cleaved-caspase-8 were purchased from Abcam, USA; antibodies against p-PERK, eIF2α and p-eIF2α were purchased from Cell Signaling Technology, USA; β-actin and horseradish peroxidase-labeled antibodies were obtained from Santa Cruz, USA. MTT assay kit was obtained from Amresco, USA.

### Collection of EC cells

Data about middle and advanced stage EC patients admitted to Fujian cancer hospital in 2013 were retrieved and analyzed. The study was approved by the Ethics Committee of Fujian Cancer Hospital affiliated with Fujian Medical University. The neoadjuvant chemotherapy (NAC) was conducted with DOX (50–60 mg/m2 D1) every 3 weeks for 2 cycles. Patients were classified as having DOX-sensitive or resistant tumors based on the outcomes of the chemotherapy they received in the first cycle if they achieved ≥ 25% or < 25% reduction in tumor dimensions by MRI, respectively. Fresh endometrial cancer tissue samples of twenty-five patients were collected for the Western blot analysis.

### Induction of DOX-resistant EC cell line

B-MD-C1 (wt) cells in the logarithmic phase were seeded in culture flasks, grown to 70% to 80% confluence, and then cultured in medium containing DOX (5 mg/L) for 1 h. The cells were washed twice in PBS and cultured in DOX-free medium. Once reaching the logarithmic phase, the cells were subcultured twice and then subjected to the DOX treatment as above for 1 to 72 h in each treatment duration, which totally lasted 8 months. The cells that sustained to grow in 5 mg/L DOX was considered tolerant and were used for tolerance assay.

Alternatively, B-MD-C1 (wt) cells in the logarithmic phase were seeded in culture flasks, grown to 70% to 80% confluence, and then cultured in medium containing DOX (0.02 μg/mL) for 24 h. After washing twice with PBS, the cells were grown in DOX-free medium to restore cell growth and then digested and passaged in medium containing 0.01 μg/mL DOX for 24 h. The process was repeated 8 times and then the cells were cultured in increasing concentration of DOX from 0.02, 0.05, 0.1, 0.5, 1, 1.5, 1.8, 2.0, 3.0, 3.5, 4.0, 4.5 to 5 mg/L for about 9 months till stable resistant cells were obtained.

### MTT assay for drug resistance

Cells were digested, diluted to 3 × 10^4^ cells/mL, and inoculated to the wells of 96 well plate (100 μL each well). The cells were cultured at 37 °C in 5% CO_2_ for 24 h, added with DOX at different concentrations and cultured for another 72 h before the MTT staining and measurements according to the manufacturer's instructions. OD values at 490 nm were recorded and used to calculate the growth inhibition and LC_50_. Resistance index (RI) was calculated as IC_50_ of resistant cells/IC_50_ of parental cells.

### Flow cytometry

The cells at a density of 2×10^5^ were added to the wells of 12-well plates, cultured for 24 h, added with DOX (5μmol/L) and cultured for another 4 h. The cells were harvested and rinsed twice with pre-chilled phosphate buffer (PBS), incubated in PBS containing 0.25% PFA for 5 min. After rinsed twice with pre-chilled phosphate buffer, the cells were reacted with primary antibodies in the dark for 30 m, washed in pre-chilled blocking buffer (PBS containing 2% FBS), and then incubated in the secondary anti-body solution for 30 m in the dark. The membrane expression levels of CRT were then determined using flow cytometer.

### Western blot analysis

EC cells were lysed in lysis buffer and extracted for total proteins or membrane proteins using cell surface protein isolation kit according to manufacturer's instructions (Haoranbio, Shanghai). After quantification using a spectrophotometer, the proteins were separated on SDS-PAGE by electrophoresis, transferred to nitrocellulose membrane, blocked at room temperature for 1 h, and stained with primary antibodies overnight at 4 °C. The membranes were then incubated with the secondary antibody and developed for immunological reactions.

### Design of siRNAs for VAMP, SNAP23, shRNA for PERK and transfection

Online software (http://rnaidesigner.thermofisher.com/rnaiexpress/design.do) was used to design small interfering RNA (siRNA) for the VAMP1 and SNAP23 genes and shRNA for PERK. The selected sequences were checked for non-homology with other genes in GenBank and synthesized at Sangon, Shanghai. siRNA sequences for VAMP1 and SNAP23 were 5-GGACAUCAUGCGUGUGAAU-3 and 5-GAGGCA GAGAAGACUUUAA-3, respectively. shRNA sequence for PERK was 5-TCGAGCGGCAACGCGTCCAGTAAGACTCCTGTTACTGGACGCGTTGCCGCTTTTT. EC cells in the logarithmic phase were inoculated into culture plates and transfected using Lipofectamine™ 2000 according to the manufacturer's instructions when the cells reached 80%~90% confluency.

### Detection of apoptosis by flow cytometry

Cells were digested and resuspended at 1×10^6^ cells/ml PBS, labeled with Annexin V and propidiumiodide (PI) following the manufacturer's instruction (Biosea Biotechnology, Beijing, China). Flow cytometry (Bection Dikinson, USA) was used to assess the apoptotic cells. The quantitation of apoptotic cells was calculated by CellQuest software.

### Induction of dendritic cells *in vitro*

Peripheral blood was collected from healthy persons and mononuclear cells were isolated using lymphocyte separation solution. After culture for 2h, adherent cells were collected and cultured in RPMI 1640 medium containing hGM-CSF, rhIL-4 at 37 °C in 5% CO_2_. The medium was refreshed every two days and the cultures were freezing-thawing pulsed with antigens (Haoranbio, Shanghai) at 20μg antigen/10^6^ dendritic cells on day 5. The surface functional markers and maturity markers (CD80, CD83 and CD86) were quantified using flow cytometer.

### *In vitro* phagocytosis

DCs loaded with autologous freezing-thawing pulsed antigens were mixed with T cells at 1: 20 ratio and incubated for 3 days. The T cells were then harvested and mixed with the wild-type ECs and drug-resistant EC cells at 10: 1 ratio for 20h after DOX induction. The viability of EC cells was assayed using the MTT method.

### Transfection of CRT overexpressing lentivirus and treatment of CRT inhibitor and ERS inducer

When reached 90% confluency, cultured drug-resistant EC cells were washed two times with PBS, digested with trypsin for 3 min and suspended in RPMI 1640 medium with 10% FBS. The cells were pelleted by centrifugation, re-suspended in RPMI 1640 medium, added to the wells of 6-well plate and cultured at 37 °C in 5% CO_2_ overnight. The adherent cells were then transfected with lentivirus overexpressing CRT, treated with ERS inducer tunicamycin (at final concentration of 100 ng/ml) and CRT inhibitor tautomycin (at final concentration of 1μg/ml). The treated cells were cultured for 24 h before harvest.

### Statistical analysis

The data were expressed as means ± standard deviation (x±s) and analyzed using SPSS 19 software. Paired comparisons were tested using the t-test and multiple group comparisons were analyzed using single factor analysis of variance (ANOVA). Values with *P* < 0.05 were considered statistically significant.

## References

[1] Humber CE, Tierney JF, Symonds RP, Collingwood M, Kirwan J, Williams C, Green JA (2007). Chemotherapy for advanced, recurrent or metastatic endometrial cancer: a systematic review of Cochrane collaboration. Ann Oncol.

[2] Gorman AM, Healy SJ, Jager R, Samali A (2012). Stress management at the ER: regulators of ER stress-induced apoptosis. Pharmacol Ther.

[3] Vannuvel K, Renard P, Raes M, Arnould T (2013). Functional and morphological impact of ER stress on mitochondria. J Cell Physiol.

[4] Liu D, Zhang M, Yin H (2013). Signaling pathways involved in endoplasmic reticulum stress-induced neuronal apoptosis. Int J Neurosci.

[5] Zitvogel L, Kepp O, Kroemer G (2010). Decoding cell death signals in inflammation and immunity. Cell.

[6] Krysko DV, Agostinis P, Krysko O, Garg AD, Bachert C, Lambrecht BN, Vandenabeele P (2011). Emerging role of damage-associated molecular patterns derived from mitochondria in inflammation. Trends Immunol.

[7] Kroemer G, Galluzzi L, Kepp O, Zitvogel L (2013). Immunogenic cell death in cancer therapy. Annu Rev Immunol.

[8] Ladoire S, Hannani D, Vetizou M, Locher C, Aymeric L, Apetoh L, Kepp O, Kroemer G, Ghiringhelli F, Zitvogel L (2014). Cell-death-associated molecular patterns as determinants of cancer immunogenicity. Antioxid Redox Signal.

[9] Bifulco G, Miele C, Di Jeso B, Beguinot F, Nappi C, Di Carlo C, Capuozzo S, Terrazzano G, Insabato L, Ulianich L (2012). Endoplasmic reticulum stress is activated in endometrial adenocarcinoma. Gynecol Oncol.

[10] Panaretakis T, Joza N, Modjtahedi N, Tesniere A, Vitale I, Durchschlag M, Fimia GM, Kepp O, Piacentini M, Froehlich KU, van Endert P, Zitvogel L, Madeo F, Kroemer G (2008). The co-translocation of ERp57 and calreticulin determines the immunogenicity of cell death. Cell Death Differ.

[11] Oyadomari S, Mori M (2004). Roles of CHOP/GADD153 in endoplasmic reticulum stress. Cell Death Differ.

[12] Harding HP, Zhang Y, Zeng H, Novoa I, Lu PD, Calfon M, Sadri N, Yun C, Popko B, Paules R, Stojdl DF, Bell JC, Hettmann T (2003). An integrated stress response regulates amino acid metabolism and resistance to oxidative stress. Mol Cell.

[13] Obeid M, Tesniere A, Ghiringhelli F, Fimia GM, Apetoh L, Perfettini JL, Castedo M, Mignot G, Panaretakis T, Casares N, Metivier D, Larochette N, van Endert P (2007). Calreticulin exposure dictates the immunogenicity of cancer cell death. Nat Med.

[14] Kopecka J, Campia I, Brusa D, Doublier S, Matera L, Ghigo D, Bosia A, Riganti C (2011). Nitric oxide and P-glycoprotein modulate the phagocytosis of colon cancer cells. J Cell Mol Med.

[15] Gu YH, Wang Y, Bai Y, Liu M, Wang HL (2017). Endoplasmic reticulum stress and apoptosis via PERK-eIF2alpha-CHOP signaling in the methamphetamine-induced chronic pulmonary injury. Environ Toxicol Pharmacol.

[16] Tao YK, Yu PL, Bai YP, Yan ST, Zhao SP, Zhang GQ (2016). Role of PERK/eIF2alpha/CHOP endoplasmic reticulum stress pathway in oxidized low-density lipoprotein mediated induction of endothelial apoptosis. Biomed Environ Sci.

[17] Ryoo HD (2016). Long and short (timeframe) of endoplasmic reticulum stress-induced cell death. FEBS J.

[18] Goo TW, Park S, Jin BR, Yun EY, Kim I, Nho SK, Kang SW, Kwon OY (2005). Endoplasmic reticulum stress response of Bombyx mori calreticulin. Mol Biol Rep.

[19] Xu FF, Liu XH (2015). Calreticulin translocation aggravates endoplasmic reticulum stress-associated apoptosis during cardiomyocyte hypoxia/reoxygenation. Chin Med J (Engl).

[20] Yang Y, Li XJ, Chen Z, Zhu XX, Wang J, Zhang LB, Qiang L, Ma YJ, Li ZY, Guo QL, You QD (2012). Wogonin induced calreticulin/annexin A1 exposure dictates the immunogenicity of cancer cells in a PERK/AKT dependent manner. PLoS One.

[21] Culina S, Lauvau G, Gubler B, van Endert PM (2004). Calreticulin promotes folding of functional human leukocyte antigen class I molecules *in vitro*. J Biol Chem.

[22] Chen C, Chang C, Su T, Hsu W, Jeng Y, Ho M, Hsieh F, Lee P, Kuo M, Lee H, Chang K (2009). Identification of calreticulin as a prognosis marker and angiogenic regulator in human gastric cancer. Ann Surg Oncol.

[23] Zhou X, Yao K, Zhang L, Zhang Y, Han Y, Liu HL, Liu XW, Su G, Yuan WZ, Wei XD, Guan QL, Zhu BD (2016). Identification of differentiation-related proteins in gastric adenocarcinoma tissues by proteomics. Technol Cancer Res Treat.

[24] Stoll G, Iribarren K, Michels J, Leary A, Zitvogel L, Cremer I, Kroemer G (2016). Calreticulin expression: Interaction with the immune infiltrate and impact on survival in patients with ovarian and non-small cell lung cancer. Oncoimmunology.

[25] Galindo I, Hernaez B, Munoz-Moreno R, Cuesta-Geijo MA, Dalmau-Mena I, Alonso C (2012). The ATF6 branch of unfolded protein response and apoptosis are activated to promote African swine fever virus infection. Cell Death Dis.

[26] Zhang L, Wang A (2012). Virus-induced ER stress and the unfolded protein response. Front Plant Sci.

[27] Panaretakis T, Kepp O, Brockmeier U, Tesniere A, Bjorklund AC, Chapman DC, Durchschlag M, Joza N, Pierron G, van Endert P, Yuan J, Zitvogel L, Madeo F (2009). Mechanisms of pre-apoptotic calreticulin exposure in immunogenic cell death. EMBO J.

[28] Krysko DV, Garg AD, Kaczmarek A, Krysko O, Agostinis P, Vandenabeele P (2012). Immunogenic cell death and DAMPs in cancer therapy. Nat Rev Cancer.

[29] Wu H, Han Y, Qin Y, Cao C, Xia Y, Liu C, Wang Y (2013). Whole-cell vaccine coated with recombinant calreticulin enhances activation of dendritic cells and induces tumour-specific immune responses. Oncol Rep.

[30] Kepp O, Semeraro M, Bravo-San Pedro J, Bloy N, Buqué A, Huang X, Zhou H, Senovilla L, Kroemer G, Galluzzi L (2015). phosphorylation as a biomarker of immunogenic cell death. Semin Cancer Biol.

[31] Mavin E, Nicholson L, Rafez Ahmed S, Gao F, Dickinson A, Wang XN (2017). Human regulatory T cells mediate transcriptional modulation of dendritic cell function. J Immunol.

[32] Liu X, Li J, Liu Y, Ding J, Tong Z, Liu Y, Zhou Y, Liu Y (2016). Calreticulin acts as an adjuvant to promote dendritic cell maturation and enhances antigen-specific cytotoxic T lymphocyte responses against non-small cell lung cancer cells. Cell Immunol.

[33] Li Y, Zeng X, He L, Yuan H (2015). Dendritic cell activation and maturation induced by recombinant calreticulin fragment 39-272. Int J Clin Exp Med.

